# Environmental impact on the temporal production of chasmogamous and cleistogamous flowers in the mixed breeding system of *Viola pubescens*

**DOI:** 10.1371/journal.pone.0229726

**Published:** 2020-03-11

**Authors:** Anne L. Sternberger, Anirudh V. S. Ruhil, David M. Rosenthal, Harvey E. Ballard, Sarah E. Wyatt

**Affiliations:** 1 Department of Environmental and Plant Biology, Ohio University, Athens, Ohio, United States of America; 2 Voinovich School of Leadership and Public Affairs, Ohio University, Athens, Ohio, United States of America; 3 Interdisciplinary Molecular and Cellular Biology Program, Ohio University, Athens, Ohio, United States of America; Indian Institute of Science, INDIA

## Abstract

*Viola pubescens* is a perennial, mixed breeding herb that produces both chasmogamous and cleistogamous flowers at different times of the season. Once bud type is specified, it does not convert from one form to the other. While temporal production of the two flowers is known to be influenced by environmental factors, the specific environmental cues that signal emergence of each flower type have not been empirically studied. To investigate the environmental parameters driving seasonal development of chasmogamous versus cleistogamous flowers, a native *V*. *pubescens* population was examined during the spring and summer of 2016 and 2017. Measurements of light quantity, canopy cover, photoperiod, temperature, soil moisture, soil pH, and the number of chasmogamous and cleistogamous buds were collected on either a weekly or biweekly basis. Independent zero-inflated negative binomial (ZINB) regressions were used to model the odds of bud production (0 versus 1 bud) and bud counts (≥ 1 bud) as a function of the environmental variables. Results of the ZINB models highlight key differences between the environmental variables that influence chasmogamous versus cleistogamous bud development and counts. In addition to the ZINB regressions, individual logistic regressions were fit to the bud data. The logistic models support results of the ZINB models and, more crucially, identify specific environmental thresholds at which each bud type is probable. Collectively, this work offers novel insight into how environmental variables shape temporal development of chasmogamous and cleistogamous flowers, suggests distinct threshold values that may aid in selectively inducing each flower type, and provides insight into how climatic change may impact mixed breeding species.

## Introduction

**Fig 1:**
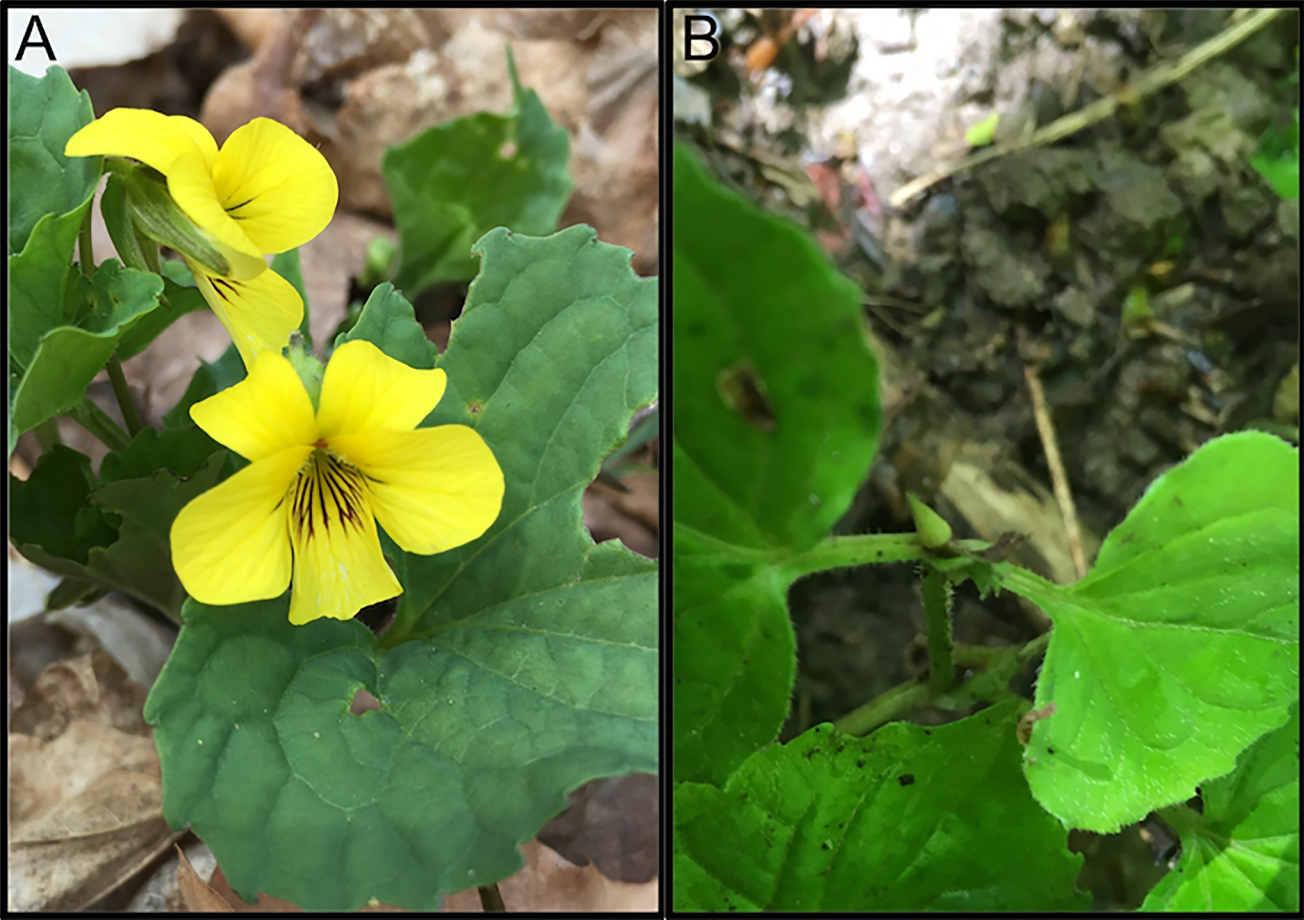
*Viola pubescens* var scabriuscula bearing A) chasmogamous and B) cleistogamous flowers. Photographs were taken over a native population in Sells Park, Athens County, Ohio.

The genus *Viola* (true violets) is well known for its large number of species, with the majority of species displaying a chasmogamous/cleistogamous mixed breeding system comprised of two distinct floral types including open, predominantly cross-pollinated chasmogamous flowers (Fig 1A), and closed, self-pollinating cleistogamous flowers (Fig 1B) [1–6]. For these mixed breeding species, chasmogamous and cleistogamous flowers can exhibit spatial separation, as seen in *Amphicarpaea bracteata*, where aerial chasmogamous flowers are isolated from subterranean cleistogamous flowers [5,7]. Or, the two flowers may develop at different times during the flowering season. This temporal separation is particularly frequent in species of temperate regions and is observed in *Viola pubescens* Aiton, an understory perennial commonly found in mesic forests of Eastern North America. *V*. *pubescens* produces chasmogamous flowers (Fig 1A) in spring and cleistogamous flowers (Fig 1B) through summer and early fall [2,8,9]. Production of the two bud types does not overlap, and once the developmental pathways of primordial buds are specified, they do not appear to be capable of converting from one floral form to the other [5,8]. Decades of physiological analyses show that most seasonal patterns of floral development are due to environmental signaling, and the mixed breeding system is also believed to be influenced by environmental factors [1,10–13]. When and whether chasmogamous or cleistogamous flowers dominate depends on growth conditions, offering an alternative mating strategy in variable environments [6,14–16].

Chasmogamous flowers possess petals and nectar to attract pollinators and produce fully formed stamens and a compound pistil with an elongate style. While chasmogamous flowers are capable of delayed self-fertilization, most species including *Viola pubescens* promote their cross-pollination [8]. In comparison, cleistogamous flowers are obligate self-pollinators with vestigial or obsolete petals, no nectar, usually reduced numbers of underdeveloped stamens (though still capable of producing a few viable pollen grains), and a reduced and commonly coiled style to effect self-pollination [16–19]. Reduced cleistogamous flowers require less energy to produce than chasmogamous flowers, and the differential cost in production of the two flowers is believed to lead to chasmogamous flowers being developmentally favored in optimal growth conditions when a surplus of resources is available [20,21]. Therefore, mixed breeding species like *V*. *pubescens* can increase genetic diversity through cross-pollination of chasmogamous flowers and also assure reproductive success via selfing of cleistogamous flowers when environmental conditions and resources are unfavorable. The mixed breeding system may also be beneficial in fragmented environments, where declines in pollinator abundance are common, and selfing via cleistogamous flowers would decrease the possibility of extinction events [22]. Thus, the chasmogamous/cleistogamous mixed breeding system is thought to provide species reproductive plasticity allowing them to adapt to heterogenous habitats and assure reproductive vigor through variable environmental and pollinator conditions [1,2,15].

Chasmogamous and cleistogamous flowers are two distinct floral forms marked by divergences in their developmental pathways [5,6,23,24]. Heterochrony-alteration in developmental timing-is proposed to be the underlying mechanism of divergence between chasmogamous and cleistogamous flowers, caused by the precocious development of cleistogamous flowers [2,24–26]. This hastened development in cleistogamous flowers is hypothesized to be exerted through early onset of meiosis and/or accelerated floral maturation [24–26]. For angiosperms, the onset and rate of flowering is largely determined by the interaction of multiple environmental cues with endogenous developmental signals [27–31]. Plants respond to these external and internal cues and “anticipate” seasonal changes by adjusting their physiology accordingly. Thus, leading to appropriate timing of the transition from the vegetative to reproductive phase and potentially chasmogamous to cleistogamous flowering when conditions are most hospitable [27,29–32].

In most temperate mixed breeding herbs, including *V*. *pubescens*, chasmogamous buds/flowers are produced in spring before the canopy closes, and cleistogamous floral organs are produced through summer and fall following canopy closure (i.e. drastic reductions in light quantity) and pollinator absence [2,6,23]. Culley [8] described that for *V*. *pubescens*, a sudden decline in light quantity causes chasmogamous flowers to abort, shortly after which plants produce cleistogamous buds instead. This transition from chasmogamous to cleistogamous budding may also reflect differing photoperiod requirements between the two flower types, as chasmogamous flowers develop close to the equinox, and cleistogamous flower are produced through the summer solstice when daylength is at its peak. Various studies also indicate that for other mixed breeding species, the ratio of chasmogamous to cleistogamous flowers produced per individual plant rises with increasing light quantity and soil fertility [1,8,10,14–16,18,33]. This correlation between flower type and light intensity is seen in *Collomia grandiflora*, where under favorable light and soil conditions, chasmogamous flowers dominate [33]. Cortes-Palomec and Ballard [1] observed a similar relationship in *Viola striata*, with chasmogamous flower production increasing as light availability increases, and Harlan [13] and Brown [12] showed positive correlations between cleistogamous flowers and poor light/soil conditions in two grass species. In contrast, Jasieniuk and Lechowicz [15] noted increases in cleistogamous flowers at higher light quantities for *Oxalis montana*.

Other studies have analyzed the influence of temperature on chasmogamous and cleistogamous flowering. In *Ceratocapnos heterocarpa* Durieu, winter temperatures were found to promote cleistogamous flowering, while warmer temperatures of spring led to greater chasmogamous flowering [34]. Similarly, for *Cardamine kokainensis*, long chilling treatments induce cleistogamous flowers while plants that do not undergo chilling produce chasmogamous and intermediate flowers [35]. For some species, the effects of soil moisture also impact the production of chasmogamous and cleistogamous flowers [12,14,16,18,36,37]. Brown [12] found that in *Stipa leucotricha* Trin. and Rupr., less available soil moisture leads to a higher percentage of cleistogamous flowers, and Waller [18] observed that high soil moisture conditions result in greater chasmogamous flower production in *Impatiens capensis*. However, the specific environmental parameter(s) and critical threshold values that drive the temporal production of chasmogamous and cleistogamous flowers remain largely unknown for most cleistogamous species. The present research focused on quantitatively analyzing how seasonal changes affect chasmogamous versus cleistogamous bud production in *V*. *pubescens* and identifying the distinct environmental factors contributing to these effects. To accomplish this work, ten plots were established over a native, unmanipulated *V*. *pubescens* population in Athens County, Ohio. For each plot, measurements of light quantity, ambient temperature, soil moisture, soil pH, and the number of chasmogamous or cleistogamous buds were collected during the spring and summer of 2016 and 2017. Photoperiod and canopy cover were also analyzed over the entire population. Field data from both years were then statistically modeled to distinguish which environmental factors are significantly associated with chasmogamous or cleistogamous bud production, the number of buds produced, and the precise environmental thresholds at which each bud type is most likely to develop.

## Results

### Chasmogamous and cleistogamous bud data

**Fig 2:**
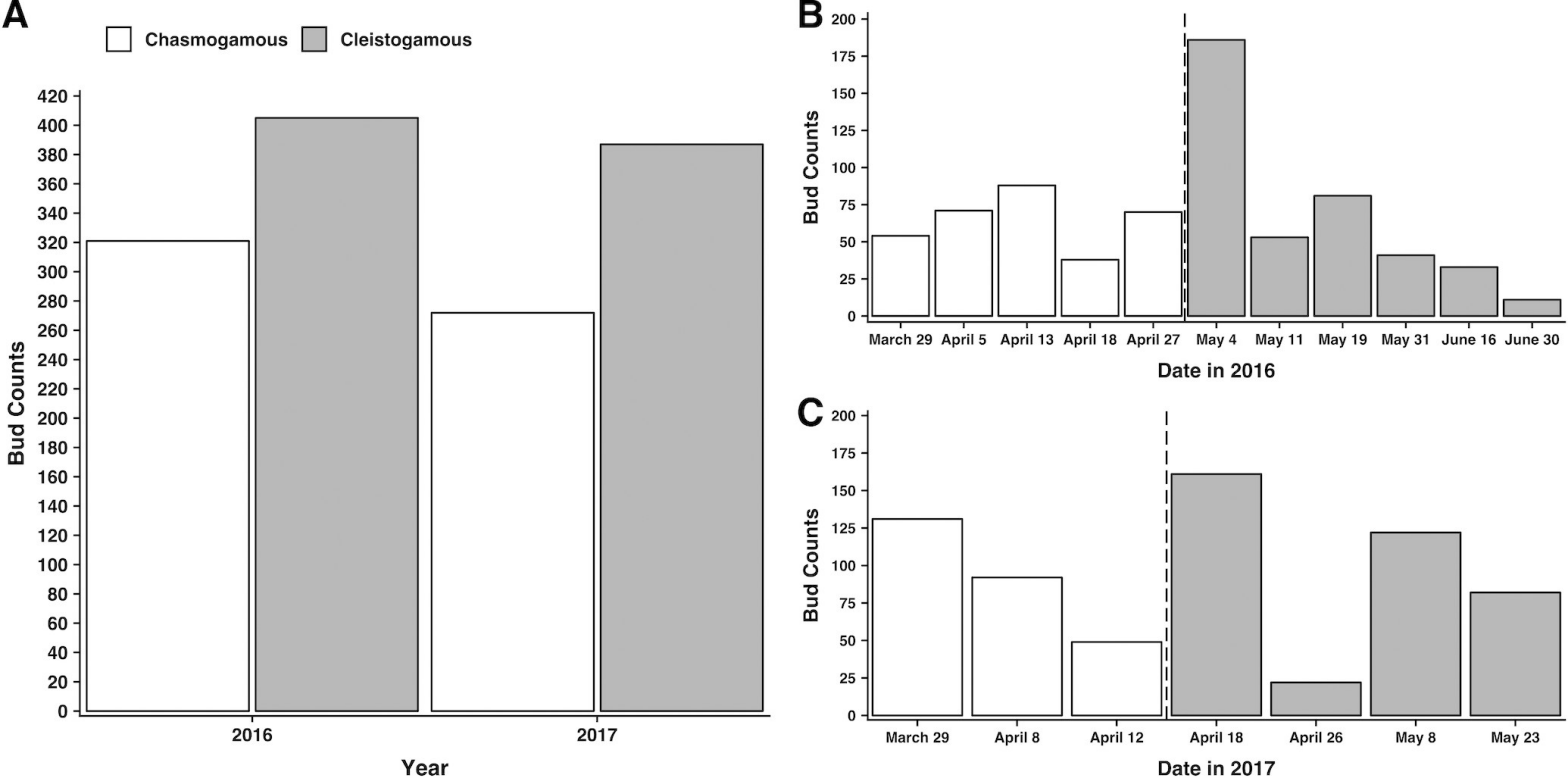
Total number of chasmogamous (white bars) and cleistogamous (gray bars) buds recorded from ten plots in A) 2016 and 2017 and at each measurement date in B) 2016 and C) 2017. Dashed vertical lines highlight bud type transition, the time at which chasmogamous budding ceased and the first cleistogamous buds were observed.

A total of 77 individual *Viola pubescens* plants were tracked for reproductive behavior during 2016 from which a total of 321 chasmogamous and 405 cleistogamous buds were recorded (Fig 2A). During 2017, 118 individuals were evaluated with 272 chasmogamous and 387 cleistogamous buds documented over the season (Fig 2A). Bud counts were collected individually from each of ten plots ~weekly and then biweekly following the transition from chasmogamous to cleistogamous budding, which will be referred to here on as bud type transition. Bud type transition occurred between April 27 and May 4, 2016 (Fig 2B) and between April 12 and April 18 in 2017 (Fig 2C). Because chasmogamous and cleistogamous buds are temporally separated, cleistogamous buds were never produced during the same measurement weeks that chasmogamous buds were present, and bud count minimums were therefore zero irrespective of bud type, plot, or year.

### Environmental variables

During 2016 and 2017, light quantity, canopy cover (i.e. canopy openness), photoperiod, ambient temperature, soil moisture, and soil pH were measured over the same plots that bud counts were collected (Table 1). Environmental variables were analyzed ~weekly—biweekly during the same days and timeframe as bud counts except for temperature, which was measured every day throughout the duration of the experiment at 15-minute (2016) and 1-hour (2017) intervals. To examine environmental influence on the development of chasmogamous versus cleistogamous buds and bud type transition, zero-inflated negative binomial (ZINB) regressions were used to model bud production (0 vs. 1 bud) and bud counts (≥ 1 bud) as a function of the combined environmental data from 2016 and 2017 (Table 2). Canopy cover (S1 Fig) was excluded from the ZINB regressions, because it was highly correlated with light quantity, and light quantity measurements better captured the light environment over individual plots and offered a larger sample size. Soil pH was also excluded from the regressions as the data were not significant for either field season (S2 Fig) and may have introduced excess noise.

**Table 1:** Environmental variables measured over a native *V*. *pubescens* population (n^1^ = 10 plots) during spring and summer of 2016 and 2017 in Sells Park, Athens County, Ohio. Environmental variables were averaged^†^ prior to calculating summary statistics.

	n^2^	Minimum	Maximum	Mean	Std. Dev.
**Variables 2016**					
Light quantity (μmol m^-2^s^-1^)	90	1.63	1,183	257.36	±314.34
Canopy openness (%)	11	2	44.19	19.86	±14.83
Photoperiod (hours)	9	12.83	14.88	13.93	±0.67
Mean temperature (°C)	90	5.08	30.38	15.91	±5.28
Soil moisture (%)	90	11	39.36	28.56	±6.54
Soil pH	70	5.12	6.70	5.84	±0.44
**Variables 2017**					
Light quantity (μmol m^-2^s^-1^)	70	4.57	1,249	333.86	±323.98
Canopy openness (%)	7	3.61	38.81	19.26	±12.31
Photoperiod (hours)	7	12.52	14.55	13.46	±0.65
Mean temperature (°C)	70	7.17	22.83	15.20	±4.39
Soil moisture (%)	70	12.60	43.20	27.50	±7.35
Soil pH	70	4.66	6.38	5.60	±0.44
**Combined data**					
Light quantity (μmol m^-2^s^-1^)	160	1.63	1,249	290.83	±320.85
Canopy openness (%)	18	2	44.19	19.67	±13.97
Photoperiod (hours)	16	12.52	14.88	13.72	±0.70
Mean Temperature (°C)	160	5.08	30.38	15.60	±4.92
Soil moisture (%)	160	11	43.20	28.09	±6.92
Soil pH	140	4.66	6.70	5.68	±0.45

^†^For light quantity and soil moisture, five measurements were taken per plot at each measurement date. Data were grouped by year, date, and plot and then averaged. Temperature was measured every day at 15-minute (2016) and 1-hour (2017) intervals, and data were also grouped by year, date, and plot before averaging. Thus, for these variables, values above reflect the min, max, mean and std. dev. calculated on the weekly averages across all plots and measurement dates. Canopy cover values (3 per measurement date) measured over the entire population were grouped by year/date and averaged. Single, daily photoperiod values were extracted from the nearest weather station for each measurement date, and one soil sample was collected per plot/measurement date. Canopy cover data were therefore grouped by year and date and soil pH data by year, date, and plot, but data were not averaged. Note that this table provides summary statistics for the averaged environmental data but does not reflect the data structure used as input for modeling (see Methods). n^2^ = number of weekly averages.

**Table 2:** Results of the zero-inflated negative binomial (ZINB) regression models predicting the odds of chasmogamous and cleistogamous buds developing and expected bud counts as a function of the averaged, z-score transformed environmental variables from 2016 and 2017.

**Chasmogamous ZINB**	*Theta = 1*.*68*, *Log-likelihood = -230 on 11 DF*					
**Binary model (0 vs. 1 bud)**	**Estimate**	**SE**	**z-value**	**p-value**	**95% CI**	**iExp(coef)**
Intercept	-0.04	0.57	-0.08	0.94	-1.16 to 1.07	1.04
Light quantity	-4.09	1.50	-2.71	0.007	-7.03 to -1.13	59.48
Photoperiod	2.77	1.21	2.29	0.02	0.40 to 5.13	0.06
Mean temperature	1.54	0.55	2.78	0.005	0.46 to 2.63	0.21
Soil moisture	-0.17	0.55	-0.30	0.76	-1.25 to 0.92	1.18
**Count model (≥1 bud)**						**Exp(coef)**
Intercept	1.77	0.23	7.83	4.78e-15	1.33 to 2.21	5.86
Light quantity	-0.23	0.13	-1.76	0.08	-0.49 to 0.03	0.79
Photoperiod	-0.45	0.23	-1.94	0.052	-0.91 to 0.005	0.63
Mean temperature	-0.07	0.11	-0.71	0.48	-0.28 to 0.13	0.93
Soil moisture	-0.07	0.14	-0.50	0.62	-0.34 to 0.20	0.93
Log(theta)	0.52	0.22	2.32	0.02	-	-
**Cleistogamous ZINB**	*Theta = 1*.*04*, *Log-likelihood = -291*.*3 on 11 DF*					
**Binary model (0 vs. 1 bud)**	**Estimate**	**SE**	**z-value**	**p-value**	**95% CIs**	**iExp(coef)**
Intercept	-0.08	0.56	-0.15	0.88	-1.19 to 1.02	1.09
Light quantity	4.21	1.84	2.30	0.02	0.61 to 7.81	0.01
Photoperiod	-2.54	1.47	-1.73	0.08	-5.42 to 0.34	12.63
Mean temperature	-2.10	1.30	-1.60	0.11	-4.61 to 0.47	7.92
Soil moisture	1.62	0.78	2.06	0.04	0.08 to 3.15	0.20
**Count model (≥1 bud)**						**Exp(coef)**
Intercept	2.98	0.29	10.25	<2e-16	2.41 to 3.55	19.69
Light quantity	1.00	0.54	1.87	0.06	-0.05 to 2.05	2.73
Photoperiod	-0.28	0.28	-1.02	0.31	-0.82 to 0.26	0.76
Mean temperature	-0.75	0.19	-3.88	1.03e-4	-1.13 to -0.37	0.47
Soil moisture	-0.11	0.13	-0.83	0.41	-0.36 to 0.15	0.90
Log(theta)	0.04	0.23	0.16	0.88	-	-

DF = degrees of freedom, SE = standard error, CI = confidence interval, iExp(coef) = inverted and exponentiated coefficients (inverted odds ratios), Exp(coef) = exponentiated count coefficients (incidence rate ratios).

Prior to modeling, environmental data were averaged. For light quantity and soil moisture, five measurements were taken per plot (n^1^ = 10) at each measurement date and averaged by plot (i.e. for each measurement date, there is one averaged light quantity and one averaged soil moisture value per plot). Temperature values were also averaged by measurement date and plot. Single, daily photoperiod values were extracted from the nearest weather station for each measurement date, and photoperiod data were therefore not averaged as with the other environmental variables. Following averaging, the environmental data were transformed into z-scores and merged by plot and date with the bud count data. Conversion to z-scores allowed for ease of interpretation of the ZINB results since for every environmental variable, a one-unit increase equaled that variable’s standard deviation. The two equations that comprise the ZINB regressions include one that models the binary distribution of zero versus non-zero bud outcomes (0 vs. ≥1 bud) and the other that models bud counts (i.e. instances of 1 or more buds). Coefficients of the ZINB binary distribution were exponentiated and inverted to represent the relative chance that a bud will be produced (i.e. 1 bud as the constant) given exposure to a particular environmental variable (Table 2). Coefficients of the count model were also exponentiated providing incidence rate ratios denoting how likely it is that one or more buds will occur given environmental conditions (Table 2).

**Fig 3:**
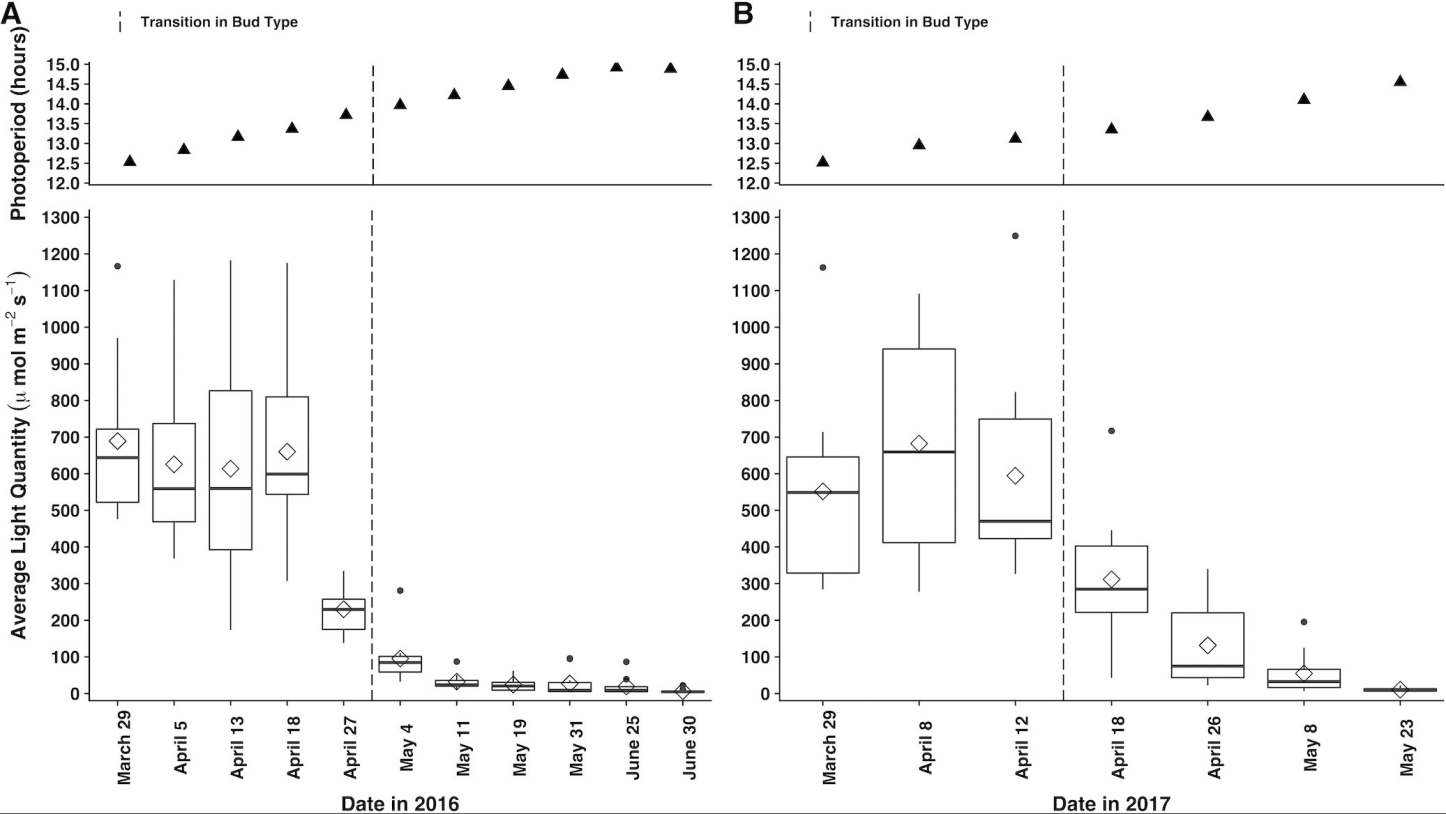
Seasonal photoperiod and averaged light quantity measured in A) 2016 and B) 2017. At each measurement date, single photoperiod values were extracted from the nearest weather station, while five measurements of light quantity were taken per plot (n^1^ = 10). For graphical representation of light quantity, data were grouped by plot and date and averaged into single, weekly values for each plot. Boxplots represent the minimum, first quartile, median, mean (◇), third quartile, and maximum calculated on the weekly averages across plots. Dashed vertical lines highlight bud type transition, the time at which chasmogamous budding ceased and the first cleistogamous buds were observed.

Light quantity, photoperiod, and mean temperature are significant in predicting whether a chasmogamous bud develops or not, whereas light quantity and soil moisture are significant in cleistogamous bud production (Table 2). Holding all other variables constant, the odds of a chasmogamous bud developing increase by a factor of 59.48 (Table 2) for every standard deviation (SD) (Table 1) increase in average light quantity. Conversely, the odds of a cleistogamous bud developing decrease by a factor of 0.01 (Table 2) for every SD (Table 1) increase in average light quantity. In 2016, bud type transition occurred between April 27 and May 4 (Fig 2B) which was accompanied by a reduction in average light levels from 229.93 μmol m^-2^s^-1^ to 95.44 μmol m^-2^s^-1^ (Fig 3A). Light quantity decreases were even more pronounced between April 18 and May 4, with a difference of 564.72 μmol m^-2^s^-1^ between means. A similar pattern was observed in 2017, with bud type transition transpiring between April 12 and April 18 (Fig 2C) and average light levels decreasing from 594.44 μmol m^-2^s^-1^ to 311.74 μmol m^-2^s^-1^ respectively (Fig 3B).

In contrast to average light quantity, the odds of a chasmogamous bud developing decrease by a factor of 0.06 (Table 2) with every SD (Table 1) increase in photoperiod. Between bud type transition weeks in 2016, photoperiod rose from 13.72 hours to 13.97 hours (Fig 3A). Like average light quantity, differences in photoperiod were most stark between April 18 and May 4 with photoperiod increasing by 0.60 hours. In 2017, photoperiod extended from 13.12 hours to 13.35 hours between transition weeks and from 12.95 hours to 13.35 hours between the second to last week of chasmogamous budding and the first cleistogamous buds (Fig 3B).

**Fig 4:**
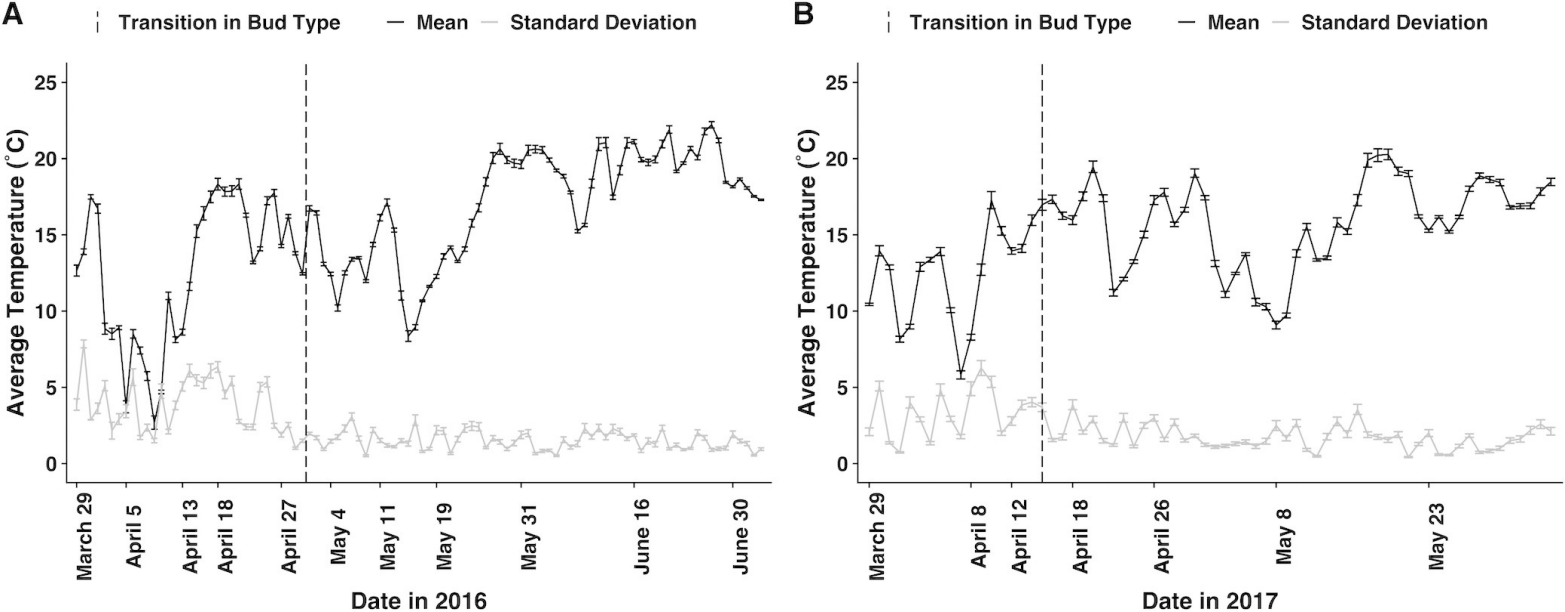
Seasonal temperature data including averaged mean and standard deviation measured in A) 2016 and B) 2017. For each of the 10 plots, temperature (°C) was measured at 15-minute (2016) and 1-hour intervals (2017). For data visualization, temperature data were grouped by measurement date and averaged to portray single, weekly observations encompassing the entire population. Error bars reflect the standard error, and dashed vertical lines highlight bud type transition, the time at which chasmogamous budding ceased and the first cleistogamous buds were observed.

For chasmogamous buds, mean temperature is also significant in predicting whether a bud develops. Odds of a chasmogamous bud developing are predicted to decrease by a factor of 0.21 (Table 2) for every unit SD (Table 1) increase in mean temperature. In 2016, mean temperature declined between the week preceding the last chasmogamous budding and bud type transition dates (Fig 4A). On the basis of the ZINB model, the odds of a chasmogamous bud developing should increase between these dates, which is inconsistent with the timing of bud type transition. However, looking at the complete temperature data instead of snapshots in time for single dates, it is evident that temperature fluctuated greatly leading up to the transition from chasmogamous to cleistogamous budding (Fig 4A). Unlike in 2016, mean temperatures in 2017 increased between the last weeks of chasmogamous budding and the first cleistogamous budding and is more compatible with the regression predictions and bud data (Fig 4B). Though, similar to 2016, temperature oscillated across these dates.

**Fig 5:**
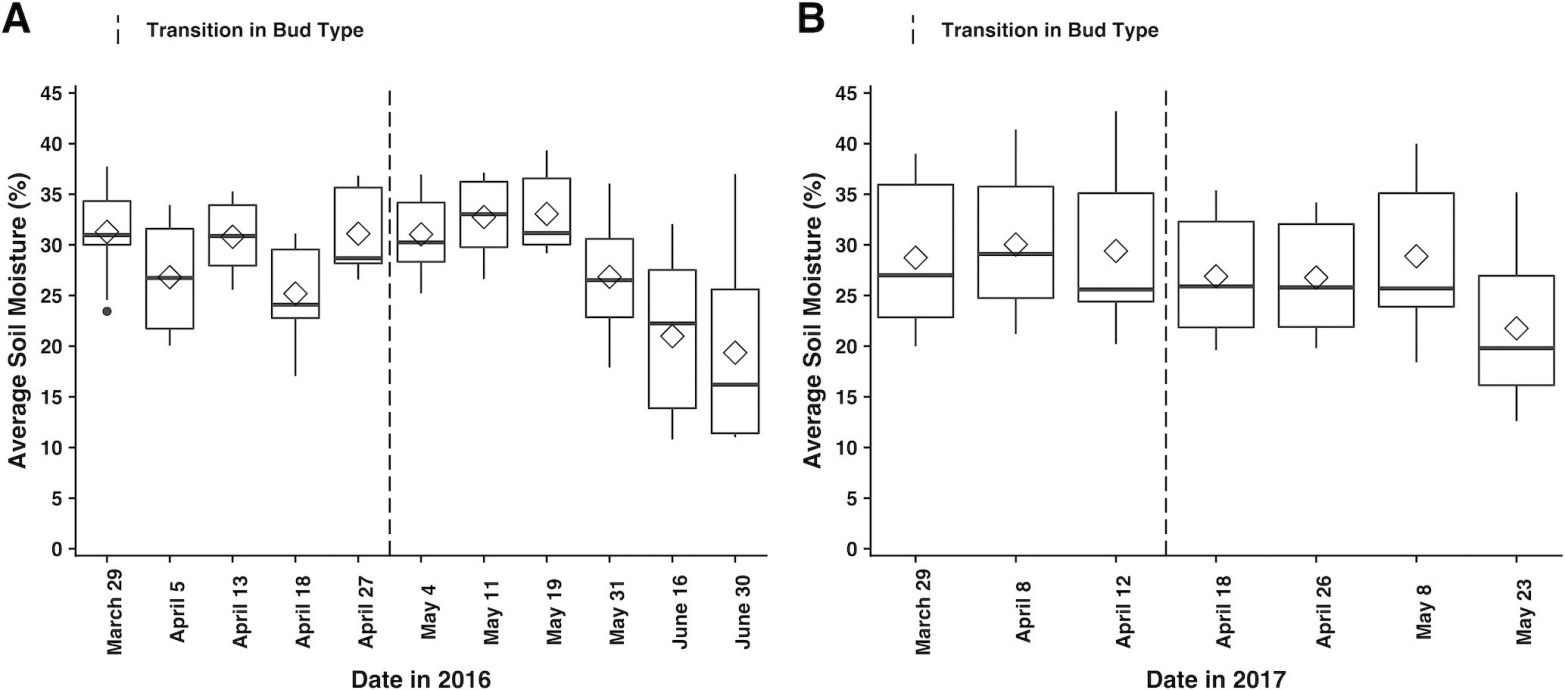
Seasonal soil moisture data measured in A) 2016 and B) 2017. From the ten plots, five measurements of soil moisture were taken per measurement date. For graphical representation, data were grouped by plot and date and averaged into single, weekly values for each plot. Boxplots represent the minimum, first quartile, median, mean (◇), third quartile, and maximum calculated on the weekly averages across plots. Dashed vertical lines highlight bud type transition, the time at which chasmogamous budding ceased and the first cleistogamous buds were observed.

Soil moisture is significant in predicting cleistogamous bud presence but is not significant in chasmogamous bud development. The odds of a cleistogamous bud developing decrease by a factor of 0.20 (Table 2) for every SD (Table 1) increase in soil moisture. On April 18, 2016, average soil moisture across plots was 25.20% (Fig 5A). On April 27, soil moisture had increased to 31.12% and then slightly decreased to 31.04% on May 4, the date cleistogamous buds were first noted. Based on these values, the odds of a cleistogamous bud developing should have decreased on May 4 in comparison to April 18. While this is inconsistent with the timing of bud type transition, the difference between average soil moisture during these weeks is 5.84%, which is less than one SD (Table 1) increase. Also, as with temperature, soil moisture varied between weeks with no clear pattern (Fig 5A). In 2017, soil moisture values were more consistent with the ZINB predictions with average soil moisture decreasing between the last dates of chasmogamous budding from 30.04% to 29.4% and to 26.90% on April 18, the date of the first recorded cleistogamous buds (Fig 5B). But again, the difference between values is less than one SD in percent soil moisture, and the data exhibit no clear seasonal pattern.

To supplement the binary ZINB models, independent logistic regressions for chasmogamous and cleistogamous buds were also fit to the averaged environmental data, and resulting coefficients were exponentiated into odds ratios. Unlike in the ZINB models, however, environmental values were not transformed into z-scores. Keeping environmental data in their original metrics allowed for estimation of specific threshold values at which chasmogamous and cleistogamous buds were predicted to develop, as a one-unit increase corresponds to a value of +1 (versus the standard deviation as in the ZINB models). For example, a one-unit increase in light equals +1 μmol m^-2^s^-1^. Similar to the inverted exponentiated coefficients of the ZINB binary models, the exponentiated coefficients of the logistic models predict the odds of bud presence (i.e. 1 bud as the constant). Results of the logistic regression for chasmogamous buds agree with the ZINB and suggest that as light quantity increases, so does the chance of a chasmogamous bud developing, and as photoperiod and mean temperature increase, chasmogamous bud likelihood decreases (Table 3). Particularly, chasmogamous bud odds are predicted to increase by a factor of 1.004 for each μmol m^-2^s^-1^ increase in light and decrease by a factor of 0.0096 with every 1 hour increase in photoperiod and by a factor of 0.84 for each 1°C increase in mean temperature.

**Table 3:** Results of the binary logistic regressions predicting the odds of chasmogamous and cleistogamous buds developing as a function of the averaged environmental variables.

**Chasmogamous**	*Null deviance = 218 on 159 DF*, *Residual deviance = 69 on 155 DF*					
	**Estimate**	**SE**	**z-value**	**p-value**	**95% CI**	**Exp(coef)**
Intercept	64.81	17.56	3.69	2.23e-4	33.97 to 103.87	1.40e+28
Light quantity	0.003	0.002	2.0	0.04	5.22e-4 to 0.01	1.004
Photoperiod	-4.64	1.24	-3.73	1.89e-4	-7.41 to -2.46	0.0096
Mean temperature	-0.18	0.07	-2.57	0.01	-0.33 to -0.05	0.84
Soil moisture	-0.01	0.06	-0.09	0.93	-0.12 to 0.11	0.99
**Cleistogamous**	*Null deviance = 222 on 159 DF*, *Residual deviance = 120 on 155 DF*					
	**Estimate**	**SE**	**z-value**	**p-value**	**95% CI**	**Exp(coef)**
Intercept	-12.06	9.46	-1.27	0.20	-31.09 to 6.37	5.79e-6
Light quantity	-0.0064	0.002	-2.99	0.003	-0.01 to -0.002	0.99
Photoperiod	0.99	0.67	1.48	0.14	-0.32 to 2.35	2.70
Mean temperature	0.08	0.05	1.53	0.13	-0.02 to 0.19	1.08
Soil moisture	-0.05	0.04	-1.36	0.17	-0.14 to 0.02	0.95

DF = degrees of freedom, SE = standard error, CI = confidence interval, Exp(coef) = exponentiated coefficients reflecting odds ratios.

**Fig 6:**
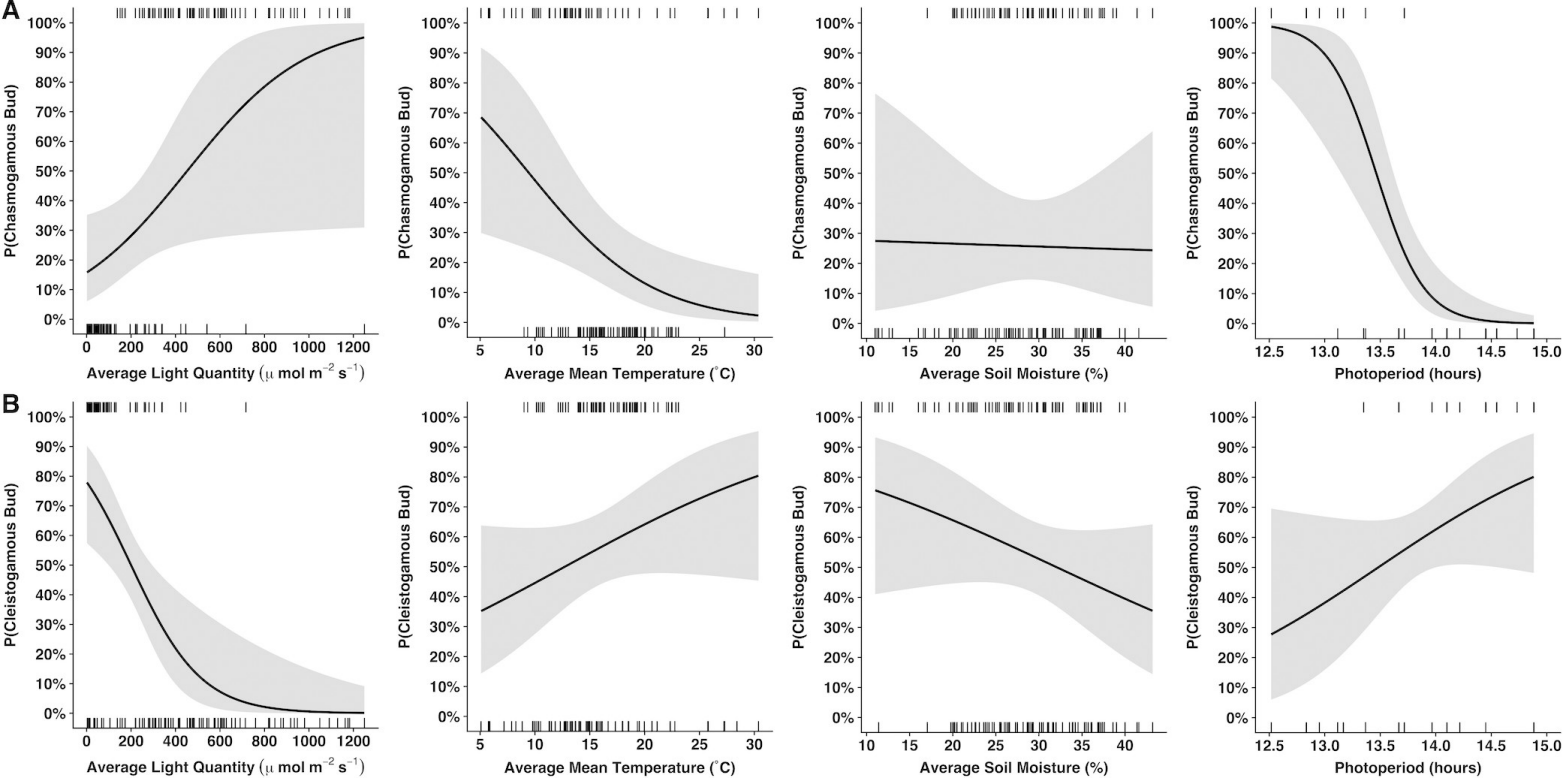
Conditional plots representing predicted bud probabilities (P) of A) chasmogamous and B) cleistogamous buds developing as each environmental variable deviates by one-unit and while holding all other variables constant at their medians. Rugs on the top of plots denote observations with positive residuals and bottom rugs reflect negative residuals.

Output from the logistic regression modeling cleistogamous buds also aligns with the ZINB results. Cleistogamous bud presence is less likely as light quantity increases, with bud odds decreasing slightly by a factor of 0.99 with every 1 μmol m^-2^s^-1^ increase (Table 3). Dissimilar to the ZINB results, the logistic regression indicates that soil moisture is not significant in predicting cleistogamous bud presence. To visualize results of the logistic regressions, odds ratios were transformed to the probability scale and conditional plots were generated reflecting chasmogamous (Fig 6A) and cleistogamous (Fig 6B) bud probabilities as environmental variables fluctuate by one-unit. The conditional plots exhibit inverse probabilities between bud types for many of the environmental variables, emphasizing that the two buds develop in response to specific and differing environmental thresholds. To validate the results of the logistic regressions, predicted bud probabilities were calculated for all measurement dates using the corresponding weekly environmental values as input (S1 Table). Bud probabilities are supported by the bud count data and the timing of bud type transitions, indicating strong model fits.

In comparison to the binary ZINB and logistic regressions which model the odds/probability of bud absence or presence, the negative binomial portion of the ZINB predicts the expected number of buds as environmental variables waver. For chasmogamous buds, only the intercept was significant in the negative binomial regression (Table 2). Cleistogamous bud counts on the other hand are indicated to be significantly impacted by mean temperature, with the number of cleistogamous buds predicted to decrease by a factor of 0.47 for every 4.92°C increase (Table 2). On May 4, 2016, the first recorded date of cleistogamous buds, mean temperature across plots was 12.44°C. Between May 4 and May 11, mean temperature ranged from 10.20°C to 16.13°C (Fig 4A). A notable decrease in bud counts occurred between these dates with 186 cleistogamous buds recorded on May 4 and 53 buds on May 11 when mean temperature was highest for this timeframe (Fig 4A). Comparable to 2016, on April 18, the first date of cleistogamous budding in 2017, mean temperature was 15.97°C (Fig 4B) and 161 cleistogamous buds were noted. On April 26, mean temperature was 17.28°C and 22 buds were observed. Between these dates, mean temperature ranged from 12.10°C to 19.50°C, one of the highest mean values for the entire field season (Fig 4B).

### Additional data explorations

From the ZINB and logistic regressions, it is apparent that differing environmental parameters significantly influence whether and how many chasmogamous or cleistogamous buds develop. To dig deeper into the regression models to identify significant differences in bud counts and environmental variables between bud types, measurement years, dates, plots, etc., nested one-way ANOVAs with all possible two-way interactions were applied to the bud count data and z-score transformed environmental variables (S2 Table). Mild significance was detected between bud counts and measurement years (S2 Table). This is likely due to the fact that buds were counted a month longer in 2016 than in 2017 (see Methods). Significant differences were also detected between bud counts and plots (S2 Table). A probable explanation is topographical differences between plots leading to differences in microhabitat. Additionally, significant differences were identified between the interaction of date and bud type on bud counts (S2 Table), which can be explained by the temporal separation of chasmogamous and cleistogamous buds.

**Fig 7:**
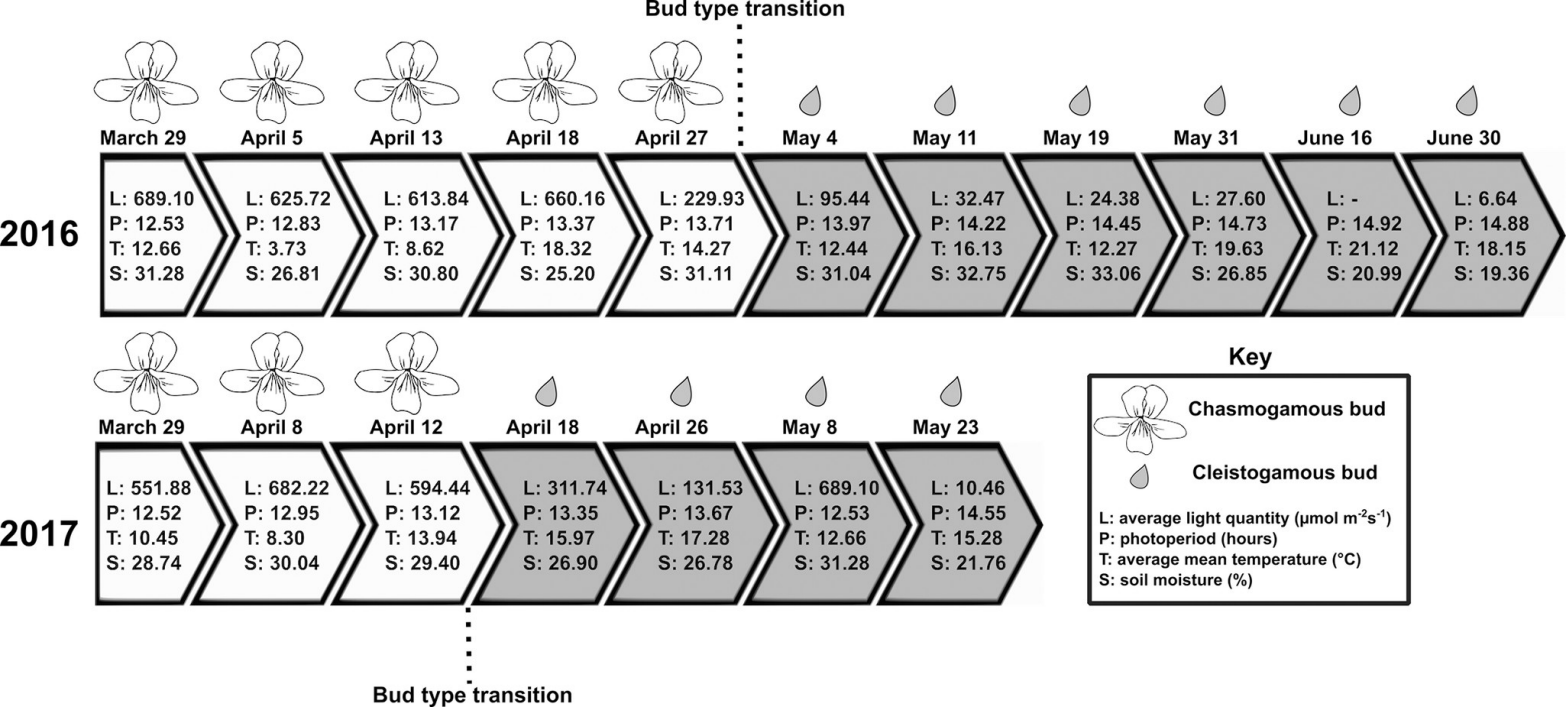
Schematic of measurement days in 2016 and 2017 with corresponding bud types and environmental values that occurred at each date. White arrows denote dates with chasmogamous buds and gray arrows are dates that cleistogamous budding was observed. L = average light quantity (μmol m^-2^s^-1^), P = photoperiod (hours), T = average mean temperature (°C), and S = average soil moisture (%).

Of particular interest in the secondary analyses were significant differences in environmental variables between measurement dates directly before and following the transition from chasmogamous to cleistogamous budding, as the environmental variables that signal for transition must occur prior to the first observed cleistogamous bud. Therefore, these specific dates are discussed in-depth below, but results of all the pairwise comparisons are provided in S3 Table (S3 Table). In 2016, bud type transition ensued between April 27 and May 4 (Fig 7), and light quantity values were similar for both dates (S3 Table). However, significant light quantity differences were found between April 18, the second to last date of chasmogamous budding and May 4, the first date cleistogamous budding was observed (S3 Table). In 2017, bud type transition occurred between April 12 and April 18 (Fig 7). Unlike in 2016, average light quantity significantly differed between transition dates as well as between the second to last date of chasmogamous budding (April 8) and first cleistogamous budding (S3 Table). Looking across both years, significant differences in average light quantity were found between year, date, plot, the interaction between year:date, and the interaction of year:plot (S2 Table). During 2016 and 2017, photoperiod steadily increased through the season, but because data was limited to daily observations, sample sizes were too small to test for differences between measurement dates.

Looking again at April 27 and May 4, bud type transition weeks in 2016 (Fig 7), there were significant differences in mean temperature between dates (S3 Table). Significant differences were even more pronounced between April 18, the date prior to the last chasmogamous budding, and May 4 (S3 Table). Similarly, significant differences in mean temperature were found between transition weeks in 2017, but temperature differences were more significant between the second to last date of chasmogamous budding and first cleistogamous buds (S3 Table). Mean temperature was comparable between years but significantly differed by date and plot (S2 Table). Percent soil moisture was also explored. For both 2016 and 2017, no significant differences in soil moisture were found between bud type transition dates or the second to last week of chasmogamous budding and the first observed cleistogamous buds (S3 Table). Finally, percent soil moisture was not significantly different between years but did exhibit significant variation between measurement dates, plots, and the interaction between year:plot and date:plot (S2 Table). Significant plot variation is again likely due to topographical differences between plots as significant differences were most prevalent between plots that were established on level ground and those located on steeper, well-drained slopes with characteristically drier soil.

## Discussion

Chasmogamous and cleistogamous buds of *Viola pubescens* develop in response to distinct light quantity values. As the average light quantity decreases, the odds of a chasmogamous bud developing decrease, while the odds of a cleistogamous bud increase. These odds and their relationship to light quantity may relate to differences in resource requirements between chasmogamous and cleistogamous flowers. As the canopy closes and plants near bud type transition, light quantity is drastically reduced in the understory. While the showy floral organs and nectar of chasmogamous flowers require large amounts of energy to produce, the miniscule size and lack of nectar in cleistogamous flowers makes them much less energetically costly [16,17]. Consequently, bud type transition may occur when light quantity drops below a critical threshold and is no longer adequate to support chasmogamous floral development. Another related but unmeasured factor that may be involved in bud type transition is pollinator presence. Like light quantity, pollinator presence is greatly reduced as the canopy closes. Cross-fertilization of chasmogamous flowers is contingent upon the availability of pollinators in early spring, while cleistogamous flowers are obligate self-fertilizers and not dependent on pollinating agents [5]. It is possible then that pollinator absence may act as the signal for bud type transition and light quantity is a confounding factor. Competition may also play a role in bud type transition. Chasmogamous flowers develop not only when light quantity and pollinator presence are high, but when there are not many other species dominating the forest floor. Without other species overcrowding them, chasmogamous flowers may have access to greater resources and be more visible to pollinators. These hypotheses may also explain the significant decrease in chasmogamous bud odds with increasing photoperiod. As the canopy closes, reductions in light quantity and pollinator presence occur simultaneously with photoperiod increase. In comparison to chasmogamous flowers, cleistogamous flowers do not require as much energy input or pollinating agents, and thus it makes sense that their buds are able to develop under low light conditions and that cleistogamous bud odds are not significantly impacted by photoperiod. The decreasing odds of chasmogamous budding with increasing photoperiod are supported by previous literature demonstrating that *V*. *fimbriatula*, *V*. *papilionaceae*, and *V*. *odorata* only produce chasmogamous flowers during short days and cleistogamous flowers under long day conditions [38,39].

For other species, both photoperiod and temperature have been implicated in dictating flower type with chasmogamous flowers observed only under short daylengths with cool temperatures, and cleistogamous flowers developing in warmer, long-day conditions [40,41]. These findings are not only consistent with the regression predictions for photoperiod, but also with the results for increasing mean temperature, which is expected to decrease the odds of chasmogamous bud development. These odds may reflect a safety mechanism to produce chasmogamous flowers when temperatures are mild and not at risk of damaging the open, exposed floral organs. The sealed nature of cleistogamous buds may reduce their risk of stress and damage under high temperatures leading to cleistogamous bud odds not being as significantly impacted by mean temperature, as the regression models suggest. Interestingly, while the odds of cleistogamous bud development are not predicted to be significantly affected by temperature, the number of buds that develop are predicted to decrease with increasing mean temperature. Although cleistogamous buds can develop under higher temperatures than chasmogamous buds, it is possible that lower temperatures are still more optimal and less stressful on the plant as a whole, promoting more numerous budding. It is also possible that the regression results for temperature are simply an artifact of the highest cleistogamous bud counts coinciding with lower mean temperatures, and other environmental variables or their interplay with temperature are more responsible for driving bud count patterns.

Whereas increasing soil moisture is not significant in predicting chasmogamous bud presence or number of buds, it is indicated by the ZINB to significantly decrease cleistogamous bud odds. However, this relationship is not observed in the bud data. Based on soil moisture averages, cleistogamous bud odds and counts should have decreased between bud type transition weeks. It is possible that soil moisture fluctuated between measurement dates and that those data would corroborate results of the ZINB. Though, this seems unlikely given soil moisture’s relatively steady state. Although soil moisture increases and decreases are observed through the season, there are no significant differences across measurement dates in 2016 (other than June 30 and dates early in the season) or 2017. Additionally, the ZINB p-value for soil moisture (0.039) is close to alpha, and no significance in soil moisture and cleistogamous bud presence was identified by the logistic regression. It is likely that soil moisture is not biologically significant to bud type transition which may be regulated by another variable(s). If soil moisture is biologically relevant, it may only be so in suboptimal conditions when cleistogamous flowers are favored and fewer resources, including soil moisture, are required.

The days preceding the last chasmogamous budding may be those responsible for driving bud type transition in *V*. *pubescens*. For light quantity and photoperiod, significant differences were found between the second to last week of chasmogamous budding and the first noted cleistogamous buds. Perhaps then there is a lag between a plant’s perception of critical environmental variables and growth responses. This is rational given the small likelihood that plants are able to perceive environmental signals, transduce/transmit those signals, and produce new, anatomically distinct buds within ~seven days. Aligning with the light quantity and photoperiod data, mean temperature in 2016 was at its maximum on the measurement date prior to the last chasmogamous budding, and in 2017, a drastic increase in mean temperature occurred between the second to last date of chasmogamous budding and first cleistogamous buds. It may be that this considerable increase in mean temperature acted as a signal for bud type transition. It is also possible that other unmeasured factors such as plant age and size play a role in driving chasmogamous versus cleistogamous development. For instance, in many taxa, plants are not capable of producing any bud type until they reach a certain age and/or density, and in some species, larger plant size is associated with chasmogamous flowering [5]. Because larger plants (and chasmogamous flowers) require greater resources, another factor associated with specifying bud type may be nutrient availability. It is important to note that these hypotheses are based on one intensively studied population of the very broad eastern North American range of *V*. *pubescens* sensu lato. Populations in other parts of the range could potentially show a somewhat different pattern of phenological shifts in chasmogamous and cleistogamous flowers, especially populations that differ in plant age/size and have different abiotic and biotic environments. For example, differences may be especially likely in the southerly populations where there are less dramatic canopy changes due to more moderate regional weather patterns. Further studies spanning additional environmental variables, *V*. *pubescens’* populations, and field seasons may help further our understanding of the environmental effects on chasmogamous/cleistogamous bud development and bud type transition in *V*. *pubescens* and other violets and temperate mixed breeding species.

## Conclusions

The present study highlights the environmental impacts of light quantity, photoperiod, temperature, and soil moisture on chasmogamous and cleistogamous bud development and the seasonal transition from chasmogamous to cleistogamous budding in a native *Viola pubescens* population. Several of the environmental variables examined show an inverse relationship between the likelihood of either bud type developing. The opposite relationships between environmental variables and bud likelihood support that chasmogamous and cleistogamous buds develop in response to distinct environmental cues. Results of this research also indicate that the environmental cues responsible for driving the transition from chasmogamous to cleistogamous budding may occur prior to the last week of chasmogamous budding. Although Lord and Mayers [42] demonstrated a two week delay between gibberellic acid treatment and the onset of chasmogamous flowering, this is the first time a delay between environmental signal perception and flowering response has been proposed for a mixed breeding species. To our knowledge, this is also the first study in a *Viola* species that has resolved specific environmental conditions at which each bud type is most probable. These data may inform future experiments wherein environmental thresholds are mimicked in growth chambers to preferentially induce each bud type outside of native conditions and verify output of the regressions. The manipulation of bud fate would also allow for further study into the genetic mechanisms responsible for chasmogamous versus cleistogamous buds. Resolving the genetic basis behind the development of specific bud types could enable the control of outcrossing and self-fertilization. Results of this research also provide novel insight into how chasmogamous and cleistogamous bud development may be affected as global climate change impacts factors like temperature and water availability. For example, with continuous temperature increases, it is likely that the timing and rate of chasmogamous and cleistogamous bud development will be altered with cleistogamous buds potentially favored.

For *V*. *pubescens* and chasmogamous/cleistogamous mixed breeding system species at large, outcrossing through chasmogamous flowers provides maximal genetic diversity to accommodate environmental stochasticity and the opportunity for reduced inbreeding depression and the removal of deleterious alleles from the population [2]. Seeds produced from cross-pollinated chasmogamous flowers may also exhibit hybrid vigor (heterosis) and increased fitness. In contrast, selfing through cleistogamy is energetically less costly and produces locally adaptive genotypes without resort to pollen vector intermediaries. The two flowers also often have different dispersal ranges, with cleistogamous seeds typically dispersed directly underneath parental plants and chasmogamous seeds dispersing greater distances [6]. In *V*. *pubescens* specifically, little is known about chasmogamous and cleistogamous seed dispersal other than seeds of both flower types are ballistically dispersed up to 5.4 meters and contain elaisomes to promote secondary ant dispersal [9,43]. Empirical studies characterizing the relative contributions of chasmogamous versus cleistogamous seeds (e.g. precise number of seeds per flower type and seed viability) are also lacking. However, Culley and Wolfe [9] demonstrated that for a *V*. *pubescens* population also located in Ohio, chasmogamous flowers produced the majority of successfully dispersed seeds with 22% of chasmogamous flowers setting seed compared to only 9% of cleistogamous flowers. In agreement, Culley [8] in a subsequent study noted similar numbers of chasmogamous and cleistogamous flowers, seeds, and seed mass produced per *V*. *pubescens* plant but that chasmogamous flowers were significantly more likely to disperse seeds. If global climate change does lead to self-fertilizing cleistogamous buds dominating and chasmogamous reproduction is reduced, there would be substantial impact on the dynamic of gene flow within *V*. *pubescens* populations. Results of the regressions herein provide a means of predicting chasmogamous and cleistogamous bud likelihoods under simulated climate conditions offering unique opportunities to forecast future, phenological patterns and population dynamics for violets and possibly other chasmogamous/cleistogamous mixed breeding species.

## Materials and methods

### Study species and field site

*Viola pubescens* Aiton, commonly known as the downy yellow violet, is a widespread, North American perennial herb. It is classified within the subsection Nudicaules of section Chamaemelanium and can be found in rich, mesic woods or sometimes meadows. *V*. *pubescens* is a nonclonal species with a rhizomatous growth habit and produces an abundance of chasmogamous and cleistogamous flowers over the growing season [44,45]. Chasmogamous flowers are produced in spring (late March-April), and cleistogamous flowers are produced in summer through the first frost in fall (May-September) [2,8,9]. To investigate the cues driving separate, seasonal production of chasmogamous and cleistogamous flowers in *V*. *pubescens*, ten plots were established within Sells Park, a mixed mesophytic forest in Athens County, Ohio, 45701 (39°20'40.6"N 82°04'31.9"W). Plots were placed along a 12 meter transect which spanned a ravine and was bisected by a small stream. Plot selection followed a stratified random design; a 1 m x 1 m PVC square was randomly tossed along both sides of the transect, and plot placement was finalized if the square landed in an area containing at least three individual *V*. *pubescens*. This process was repeated until ten plots were assigned. Corners of the 1 m x 1 m plots were marked with rebar and flags indicating plot number. Plots were established on level ground with the exception of three which were located on steep, well-drained slopes. The canopy over the plots is dominated by *Fagus grandifolia* (American beech) and *Acer saccharum* (sugar maple). In addition to these species, *Liriodendron tulipifera* (tulip poplar), *Quercus prinus* (chestnut oak), *Platanus occidentalis* (American sycamore), and *Carya spp*. (hickory) grow at the outskirts of the canopy. The shrub community is primarily *Lindera benzoin* (spicebush), *Asimina triloba* (pawpaw), *Vitis spp*. (grapevine), and *Verbesina alternifolia* (wingstem). The understory herbs/ferns that grow directly within or around plots include *Trillium grandiflorum* (white trillium), *Podophyllum peltatum* (mayapple), *Persicaria virginiana* (jumpweed), *Galium spp*. (bedstraw), *Sanguinaria canadensis* (bloodroot), and *Polystichum acrostichoides* (Christmas fern). Permits were not required to conduct research in the public park, and no protected species were sampled.

### Sampling of reproductive and environmental data

Field observations were conducted during chasmogamous and cleistogamous floral development of 2016 and 2017. For each of the ten plots, the number of chasmogamous or cleistogamous buds, flowers, and fruits were counted initially on a weekly basis and then every two weeks following the transition from chasmogamous to cleistogamous budding. The buds, flowers, and fruits of individual plants were tagged with unique identifiers and tracked across the season to gather accurate counts of newly developed reproductive structures between each measurement date. Bud, flower, and fruit counts were collected from each plot between 10:30 am– 12 pm on days with low to no cloud cover to ensure consistent light quantity sampling across measurement dates. Consequently, measurements were not always weekly (or biweekly following bud type transition), as days with high cloud cover or rain were bypassed. In 2016, environmental conditions and reproductive counts were evaluated from March 29 –June 30, while in 2017, they were surveyed from March 29 –May 23. The survey season in 2017 was a month shorter than in 2016 due to herbivory that decimated plants in multiple plots. Further data collected from the disturbed plots would not have reflected true bud counts as a factor of the environmental variables.

Light quantity (μmol m^-2^s^-1^) was assessed via a LI191R Line Quantum Sensor (LI-COR Biosciences, Inc.), with five measurements (corners and center) taken per plot at each measurement date. To further characterize the light environment, hemispherical photographs of the canopy were taken from the middle of the transect using Regent Instrument’s WinSCANOPY™ DSLR Compact System with NorthFinder, self-leveling O-mount, Canon EOS REBEL T1i, and a calibrated, 180° fisheye lens. Three photographs were taken per measurement date. Photographs were analyzed using WinSCANOPY™ image software to identify light availability values as percent canopy openness. Photoperiod (i.e. daylength) was extracted from the Mid-Ohio Valley Regional Airport station (Elev 0 39.29 °N, 81.53 °W) via the Historical Weather database in Weather Underground (https://www.wunderground.com/history/). To record temperature, two Onset^®^ HOBO^®^ pendant temperature loggers were hung at opposite sides of the transect, and Thermochron^®^ iButton^®^ loggers were placed at the center of each plot. During the 2016 field season, temperature was measured in fifteen minute increments which quickly maxed out the loggers’ memory capacity. To circumvent this during the 2017 season, temperature was measured hourly. Percent soil moisture was analyzed using a Hydrosense II (Campbell Scientific, Inc.), with five measurements (corners and center) collected per plot. To analyze soil pH, soil cores were collected from the center of each plot on a biweekly basis in 2016. Initial explorations of the 2016 data lead us to modify the soil measurement cycle to a weekly frequency in 2017 since that would allow for a richer dataset. Soil slurries were made using the cores and distilled water, and slurry pH was determined using a standardized pH probe.

### Statistical analyses

All statistical analyses were conducted in R (v.3.4.3). Light quantity, temperature, and soil moisture data were grouped by year, date, and plot number and then averaged to characterize weekly plot values (i.e. each of the ten plots has one averaged light quantity value, one averaged temperature value, and one averaged soil moisture value per measurement date in 2016 and 2017). Because canopy cover was measured over the entire population and not individual plots, canopy cover data was grouped only by year and date before averaging. This averaging circumvents any noise introduced by variation in when environmental measurements were recorded during a week. For pH analyses, one soil sample was collected per plot at each measurement date, and for photoperiod, single daily values were available from public weather repositories. Thus, soil pH data was grouped by year, date, and plot and photoperiod data by year and date, but data were not averaged prior to modeling. Summary statistics for the entire, unaveraged environmental data are available in S4 Table.

Bud counts and environmental data were then merged by date and plot into a single dataset, and environmental variable measurements were converted to z-scores to standardize data for easier comparison and interpretation. Bud count data were used for statistical analyses (instead of flowers and/or fruits) because using flower and/or fruit data may not have been representative of the early environmental signaling that dictates chasmogamous versus cleistogamous flowering (i.e. primordial bud type). Due to over-dispersion, no doubt in part due to the excess zeros in the data (e.g. there were zero cleistogamous buds on all the days that chasmogamous buds were present and vice versa), we relied on independent zero-inflated negative binomial regressions to model chasmogamous and cleistogamous bud type production as a function of the specified environmental variables. For both bud types, we also fit Poisson, negative binomial, and zero-inflated Poisson models before settling on the zero-inflated negative binomial models. Vuong’s non-nested test [46] was utilized to compare the Poisson and negative binomials to their zero-inflated counterparts. Once the zero-inflated models were found to better fit the data, a likelihood ratio test [47] was used to compare the zero-inflated Poisson regressions to the zero-inflated negative binomials. We also utilized the Akaike information criterion [48] for comparing across regression models. These tests and comparisons of model fits justified the use of the zero-inflated negative binomial regression for modeling both the chasmogamous and cleistogamous bud data with the environmental variables (S5 Table). Canopy cover (S1 Fig) was excluded from the ZINB regressions because of its high correlation with light quantity (r = 0.73), and average light measurements via the LI191R Line Quantum Sensor more quantitatively characterized the light environment and provided a larger sample size. Soil pH was also withheld from the ZINB regressions to limit excess noise as the data were not statistically or biologically significant across dates for either field season (S2 Fig) and if included drastically reduced model sample size due to the biweekly measurement frequency in 2016. For temperature, we included the averaged, weekly plot means in the zero-inflated negative binomial models, because we felt they would sufficiently capture biological variability, and the average maximum and minimum temperatures were too highly correlated with mean temperature (r = 0.83 and r = 0.69 respectively). Resulting coefficients for significant environmental variables from both the binary and count portions of the zero-inflated negative binomials were exponentiated into inverted odds and incidence rate ratios.

In addition to the zero-inflated negative binomial regressions, binary logistic regression models were also estimated for both chasmogamous and cleistogamous bud counts wherein the outcome was coded 0 for no buds versus 1 for instances of one or more buds. This secondary analysis was motivated by the desire to identify bud-generating threshold values of the environmental variables, as the output of zero-inflated negative binomials cannot be visualized using standard regression packages. Null and deviance residuals of the logistic regressions indicated a better fit than the null model for both chasmogamous (p = 3.06e-30) and cleistogamous (p = 1.98e-20) bud data. For interpretation of the logistic regressions, coefficients were exponentiated into odds ratios. For data visualization purposes, odds ratios were transformed to the probability scale via an inverse logistic transformation, and conditional plots were generated using the visreg package in R [49]. To support results of the logistic regressions, the predict function in R was used to calculate predicted bud probabilities at specific measurement dates using the corresponding median values of environmental variables (S1 Table). Finally, while the zero-inflated negative binomial and logistic regressions indicated which environmental variables were significant in estimating bud counts and thresholds at which 0 vs ≥ 1 chasmogamous and cleistogamous buds were more likely to occur, there was no way to further investigate significant variables without falling prey to multiple comparisons. Thus, for the bud count data and each of the significant environmental variables identified by the zero-inflated negative binomial regressions, we used nested one-way analyses of variance (ANOVAs) with a significance level of α = 0.05 to test for differences between plots, measurement weeks, years, and all possible two-way interactions. First, we conducted the usual tests for normality and equality of variance using the Shapiro-Wilk [50] and Levene’s tests [51] respectively. While the bud counts and majority of the environmental data did not meet normality or equal variance assumptions, this was expected with our seasonal data and was not a matter for concern given that the ANOVA is robust to reasonable violations of normality [52]. Non-parametric tests, such as the Kruskal-Wallis, may have resulted in loss of precision (e.g. using ranked vs. untransformed outcome data) and/or power. Post hoc tests were conducted on environmental variables with significant ANOVA findings using pairwise comparisons. Due to the problems with using multiple comparisons, we opted for a more stringent Bonferroni p-adjustment method [53–55]. The majority of figures were created using the ggplot2 package in R [56].

## Supporting information

S1 Fig Seasonal canopy cover data measured in A) 2016 and B) 2017. Dashed vertical lines highlight bud type transition, the time at which chasmogamous budding ceased and the first cleistogamous buds were observed.
(TIF)

S2 Fig Soil pH data measured in A) 2016 and B) 2017. Significant differences in soil pH between measurement dates were evaluated using one-way analyses of variance (ANOVAs) with a significance level of α = 0.05.
(TIF)

S1 Table The probability (P) of chasmogamous and cleistogamous buds developing at each measurement date in 2016 and 2017 based on the corresponding environmental values for those dates. Probabilities were calculated using output of the logistic regressions and the predict function in R. Dashed horizontal lines highlight bud type transition, the time at which chasmogamous budding ceased and the first cleistogamous buds were observed.
(PDF)

S2 Table Secondary analyses used to investigate differences in bud counts and environmental variables between plots, measurement weeks (Date), and years via one-way analyses of variance (ANOVAs) with a significance level of α = 0.05. Cells shaded in gray highlight significant comparisons.
(PDF)

S3 Table Results of pairwise comparisons investigating significant differences in average light quantity, mean temperature, and soil moisture between measurement dates in 2016 and 2017. Values within cells reflect p-values. Values surrounded by dashed boxes correspond to p-values for comparisons of bud type transition dates (i.e. the last date of chasmogamous budding and the date the first cleistogamous buds were observed), and bolded values correspond to p-values for the second to last date of chasmogamous budding and date of the first cleistogamous buds.
(PDF)

S4 Table Environmental variables measured over a native *V*. *pubescens population* (n^1^ = 10 plots) during spring and summer of 2016 and 2017 in Sells Park, Athens County, Ohio. n^2^ = number of raw observations.
(PDF)

S5 Table Model comparison of Poisson, negative binomial, zero-inflated Poisson, and zero-inflated negative binomial regressions used to model chasmogamous and cleistogamous bud data as a function of the environmental variables. Regression models were compared using Vuong’s non-nested test, the likelihood ratio test, and the Akaike information criterion.
(PDF)
