# Supplementary figures and images for: Environmental impact on the temporal production of chasmogamous and cleistogamous flowers in the mixed breeding system of Viola pubescens
Source: PLoS One. 2020 Mar 11;15(3):e0229726. doi: 10.1371/journal.pone.0229726 (PMC7065761; doi:10.1371/journal.pone.0229726)

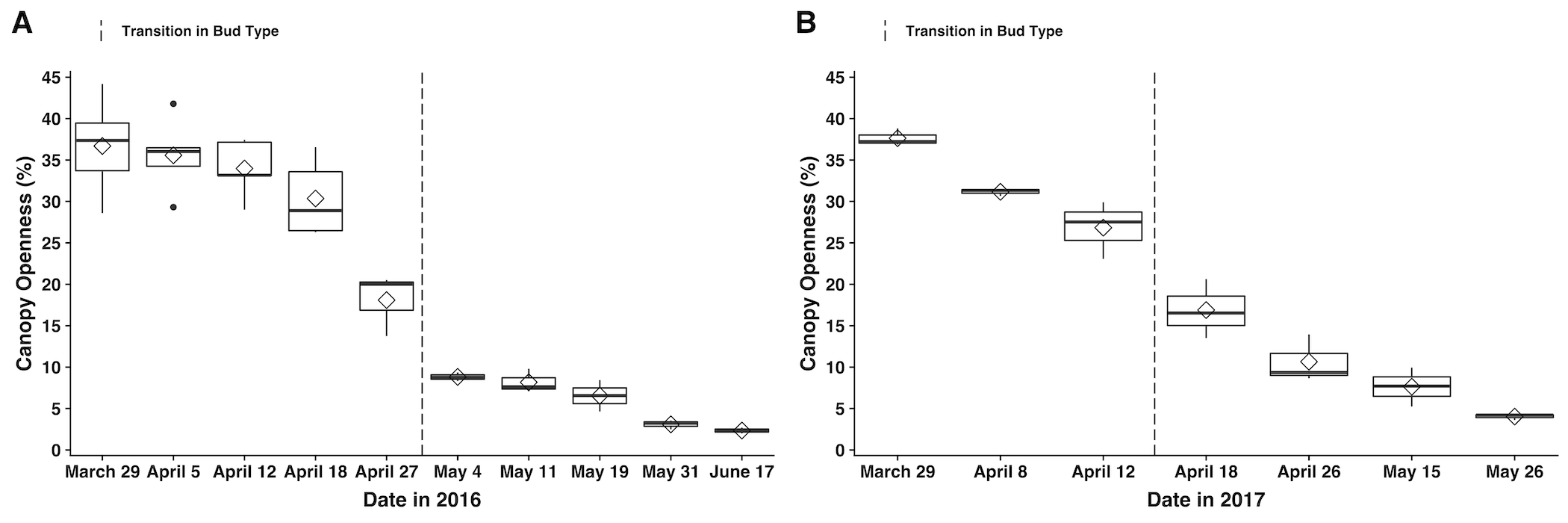

Supplement: S1 Fig — Dashed vertical lines highlight bud type transition, the time at which chasmogamous budding ceased and the first cleistogamous buds were observed. (TIF) [file pone.0229726.s001.tif]

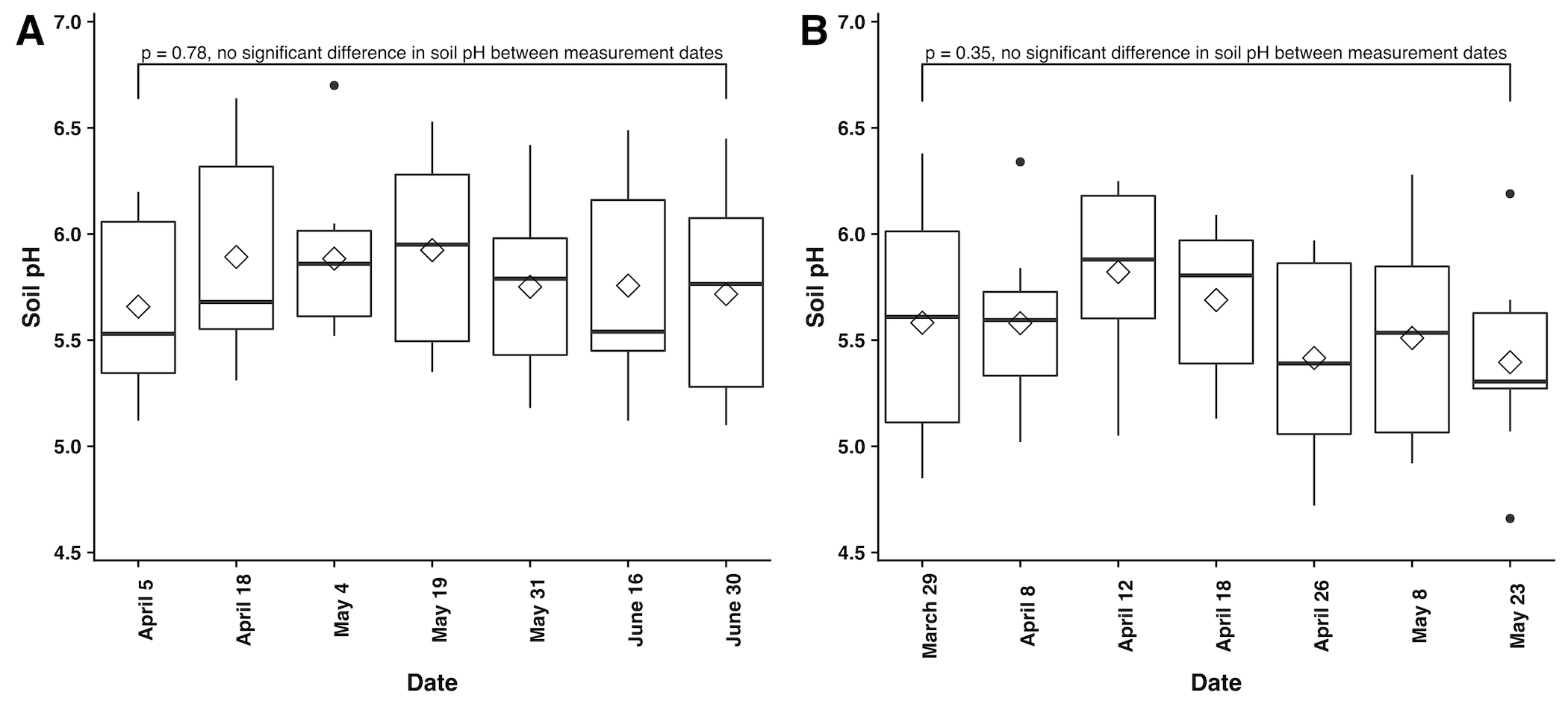

Supplement: S2 Fig — Significant differences in soil pH between measurement dates were evaluated using one-way analyses of variance (ANOVAs) with a significance level of α = 0.05. (TIF) [file pone.0229726.s002.tif]
