# Supplementary material for: Environmental impact on the temporal production of chasmogamous and cleistogamous flowers in the mixed breeding system of Viola pubescens
Source: PLoS One. 2020 Mar 11;15(3):e0229726. doi: 10.1371/journal.pone.0229726 (PMC7065761; doi:10.1371/journal.pone.0229726)
Supplement: S1 Table — Probabilities were calculated using output of the logistic regressions and the predict function in R. Dashed horizontal lines highlight bud type transition, the time at which chasmogamous budding ceased and the first cleistogamous buds were observed. (PDF) [file pone.0229726.s003.pdf]

	P(chasmogamous bud)	P(cleistogamous bud)	Bud type present
**Dates 2016**			
March 29	100%	1%	chasmogamous
April 5	99%	2%	chasmogamous
April 13	95%	5%	chasmogamous
April 18	51%	15%	chasmogamous
April 27	38%	40%	chasmogamous
May 4	7%	70%	cleistogamous
May 11	1%	81%	cleistogamous
May 19	0.88%	82%	cleistogamous
May 31	0.07%	94%	cleistogamous
June 16	0.02%	96%	cleistogamous
June 30	0.04%	96%	cleistogamous
**Dates 2017**			
March 29	100%	2%	chasmogamous
April 8	99%	2%	chasmogamous
April 12	92%	12%	chasmogamous
April 18	50%	42%	cleistogamous
April 26	10%	79%	cleistogamous
May 8	7%	77%	cleistogamous
May 23	0.33%	93%	cleistogamous
