# Supplementary material for: Environmental impact on the temporal production of chasmogamous and cleistogamous flowers in the mixed breeding system of Viola pubescens
Source: PLoS One. 2020 Mar 11;15(3):e0229726. doi: 10.1371/journal.pone.0229726 (PMC7065761; doi:10.1371/journal.pone.0229726)
Supplement: S2 Table — Cells shaded in gray highlight significant comparisons. (PDF) [file pone.0229726.s004.pdf]

Comparison	ANOVA p-value
**Bud Counts**	
Year	0.046
Date	0.060
Bud type	0.107
Plot	2.79e-8
Year:Date	0.854
Year:Bud type	0.531
Year:Plot	0.838
Date:Bud type	1.21e-10
Date:Plot	0.980
Bud type:Plot	0.914
**Average Light Quantity**	
Year	0.002
Date	<2.2e-16
Plot	0.008
Year:Date	0.001
Year:Plot	0.005
Date:Plot	0.156
***Photoperiod**	
Year	0.22
**Mean Temperature**	
Year	0.182
Date	3.31e-7
Plot	0.026
Year:Date	0.094
Year:Plot	0.579
Date:Plot	0.482
**Percent Soil Moisture**	
Year	0.080
Date	5.31e-10
Plot	<2.2e-16
Year:Date	0.214
Year:Plot	0.047
Date:Plot	0.002

*Photoperiod sample sizes were too small to meet ANOVA assumptions, and a Kruskal-Wallis test with a significance level of $\alpha = 0.05$ was used instead.
