# Supplementary material for: Environmental impact on the temporal production of chasmogamous and cleistogamous flowers in the mixed breeding system of Viola pubescens
Source: PLoS One. 2020 Mar 11;15(3):e0229726. doi: 10.1371/journal.pone.0229726 (PMC7065761; doi:10.1371/journal.pone.0229726)
Supplement: S3 Table — Values within cells reflect p-values. Values surrounded by dashed boxes correspond to p-values for comparisons of bud type transition dates (i.e. the last date of chasmogamous budding and the date the first cleistogamous buds were observed), and bolded values correspond to p-values for the second to last date of chasmogamous budding and date of the first cleistogamous buds. (PDF) [file pone.0229726.s005.pdf]

## Light quantity

**Date 2016**

| | March 29 | April 5 | April 13 | April 18 | April 27 | May 4 | May 11 | May 19 | May 31 |
|---|---|---|---|---|---|---|---|---|---|
| April 5 | 1.00 | - | - | - | - | - | - | - | - |
| April 13 | 1.00 | 1.00 | - | - | - | - | - | - | - |
| April 18 | 1.00 | 1.00 | 1.00 | - | - | - | - | - | - |
| April 27 | 1.3e-6 | 4.7e-5 | 8.9e-5 | 6.8e-6 | - | - | - | - | - |
| May 4 | 3.5e-10 | 1.8e-8 | 3.7e-8 | **2.1e-9** | 1.00 | - | - | - | - |
| May 11 | 6.6e-12 | 3.6e-10 | 7.5e-10 | 4.1e-11 | 0.47 | 1.00 | - | - | - |
| May 19 | 3.9e-12 | 2.2e-10 | 4.5e-10 | 2.5e-11 | 0.35 | 1.00 | 1.00 | - | - |
| May 31 | 4.8e-12 | 2.6e-10 | 5.6e-10 | 3.0e-11 | 0.39 | 1.00 | 1.00 | 1.00 | - |
| June 30 | 1.3e-12 | 7.0e-11 | 1.5e-10 | 8.0e-11 | 0.18 | 1.00 | 1.00 | 1.00 | 1.00 |

**Date 2017**

| | March 29 | April 8 | April 12 | April 18 | April 26 | May 8 |
|---|---|---|---|---|---|---|
| April 8 | 0.89 | - | - | - | - | - |
| April 12 | 1.00 | 1.00 | - | - | - | - |
| April 18 | 0.01 | **8.5e-7** | 4.0e-4 | - | - | - |
| April 26 | 1.8e-8 | 2.9e-13 | 5.5e-10 | 0.11 | - | - |
| May 8 | 3.0e-11 | 3.0e-16 | 7.6e-13 | 0.002 | 1.00 | - |
| May 23 | 6.5e-13 | <2e-16 | 1.5e-14 | 1.0e-4 | 1.00 | 1.00 |

## Mean temperature

**Date 2016**

| | March 29 | April 5 | April 13 | April 18 | April 27 | May 4 | May 11 | May 19 | May 31 |
|---|---|---|---|---|---|---|---|---|---|
| April 5 | <2e-16 | - | - | - | - | - | - | - | - |
| April 13 | 4.4e-7 | <2e-16 | - | - | - | - | - | - | - |
| April 18 | 3.0e-12 | <2e-16 | <2e-16 | - | - | - | - | - | - |
| April 27 | 0.58 | <2e-16 | <2e-16 | <2e-16 | - | - | - | - | - |
| May 4 | 1.00 | <2e-16 | 8.8e-16 | **<2e-16** | 5.7e-5 | - | - | - | - |
| May 11 | 1.6e-5 | <2e-16 | <2e-16 | 3.2e-6 | 3.7e-5 | 2.7e-15 | - | - | - |
| May 19 | 1.00 | <2e-16 | 8.8e-15 | <2e-16 | 7.0e-6 | 1.00 | 2.6e-16 | - | - |
| May 31 | 2.5e-16 | <2e-16 | <2e-16 | 0.03 | <2e-16 | <2e-16 | 3.0e-13 | <2e-16 | - |
| June 30 | 7.5e-12 | <2e-16 | <2e-16 | 1.00 | <2e-16 | <2e-16 | 1.4e-5 | <2e-16 | 0.01 |

**Date 2017**

| | March 29 | April 8 | April 12 | April 18 | April 26 | May 8 |
|---|---|---|---|---|---|---|
| April 8 | 2.4e-5 | - | - | - | - | - |
| April 12 | 5.9e-16 | 1.0e-4 | - | - | - | - |
| April 18 | <2e-16 | **4.8e-13** | 0.003 | - | - | - |
| April 26 | <2e-16 | 2.8e-13 | 0.002 | 1.00 | - | - |
| May 8 | 1.00 | 1.4e-5 | 2.9e-16 | <2e-16 | <2e-16 | - |
| May 23 | 6.8e-12 | 0.07 | 1.00 | 2.7e-6 | 1.8e-6 | 3.4e-12 |

## Soil moisture

**Date 2016**

| | March 29 | April 5 | April 13 | April 18 | April 27 | May 4 | May 11 | May 19 | May 31 |
|---|---|---|---|---|---|---|---|---|---|
| April 5 | 1.00 | - | - | - | - | - | - | - | - |
| April 13 | 1.00 | 1.00 | - | - | - | - | - | - | - |
| April 18 | 0.46 | 1.00 | 0.80 | - | - | - | - | - | - |
| April 27 | 1.00 | 1.00 | 1.00 | 0.47 | - | - | - | - | - |

| | | | | | | | | | |
|---|---|---|---|---|---|---|---|---|---|
| May 4 | 1.00 | 1.00 | 1.00 | **0.61** | 1.00 | - | - | - | - |
| May 11 | 1.00 | 0.55 | 1.00 | 0.07 | 1.00 | 1.00 | - | - | - |
| May 19 | 1.00 | 0.38 | 1.00 | 0.05 | 1.00 | 1.00 | 1.00 | - | - |
| May 31 | 1.00 | 1.00 | 1.00 | 1.00 | 1.00 | 1.00 | 0.57 | 0.40 | - |
| June 30 | 5.0e-4 | 0.32 | 0.001 | 1.00 | 7.0e-4 | 8.0e-4 | 4.7e-5 | 2.8e-5 | 0.31 |

**Date 2017**

| | March 29 | April 8 | April 12 | April 18 | April 26 | May 8 |
|---|---|---|---|---|---|---|
| April 8 | 1.00 | - | - | - | - | - |
| April 12 | 1.00 | 1.00 | - | - | - | - |
| April 18 | 1.00 | **1.00** | 1.00 | - | - | - |
| April 26 | 1.00 | 1.00 | 1.00 | 1.00 | - | - |
| May 8 | 1.00 | 1.00 | 1.00 | 1.00 | 1.00 | - |
| May 23 | 0.04 | 0.006 | 0.02 | 0.47 | 0.53 | 0.04 |
