# Supplementary material for: Environmental impact on the temporal production of chasmogamous and cleistogamous flowers in the mixed breeding system of Viola pubescens
Source: PLoS One. 2020 Mar 11;15(3):e0229726. doi: 10.1371/journal.pone.0229726 (PMC7065761; doi:10.1371/journal.pone.0229726)
Supplement: S4 Table — n2 = number of raw observations. (PDF) [file pone.0229726.s006.pdf]

	n^2	Minimum	Maximum	Mean	Std. Dev.
**Variables 2016**					
Light quantity (µmol $m^{-2}s^{-1}$)	1,100	1.37	1,310	274.97	±343.39
Canopy openness (%)	44	2	44.19	19.86	±14.83
Photoperiod (hours)	9	12.83	14.88	13.93	±0.67
Temperature (˚C)	96,290	-4	35.65	15.77	±5.24
Soil moisture (%)	1,100	9.2	44	28.11	±7.28
Soil pH	70	5.12	6.70	5.84	±0.44
**Variables 2017**					
Light quantity (µmol $m^{-2}s^{-1}$)	700	3.78	1,430	333.86	±347.34
Canopy openness (%)	21	3.61	38.81	19.26	±12.31
Photoperiod (hours)	7	12.52	14.55	13.46	±0.65
Temperature (˚C)	18,578	0	31.5	14.87	±4.12
Soil moisture (%)	700	12	48	27.5	±7.74
Soil pH	70	4.66	6.38	5.55	±0.44
