# Supplementary material for: Environmental impact on the temporal production of chasmogamous and cleistogamous flowers in the mixed breeding system of Viola pubescens
Source: PLoS One. 2020 Mar 11;15(3):e0229726. doi: 10.1371/journal.pone.0229726 (PMC7065761; doi:10.1371/journal.pone.0229726)
Supplement: S5 Table — Regression models were compared using Vuong’s non-nested test, the likelihood ratio test, and the Akaike information criterion. (PDF) [file pone.0229726.s007.pdf]

Vuong's non-nested test: Poisson vs zero-inflated Poisson

Chasmogamous Bud Data			
	z-statistic	*H_A*	*p-value*
Raw	-3.09	model 2 > model 1	1.0e-3
AIC-corrected	-2.95	model 2 > model 1	1.6e-3
BIC-corrected	-2.74	model 2 > model 1	3.1e-3

Cleistogamous Bud Data			
	z-statistic	*H_A*	*p-value*
Raw	-4.51	model 2 > model 1	3.27e-6
AIC-corrected	-4.44	model 2 > model 1	4.40e-6
BIC-corrected	-4.34	model 2 > model 1	6.89e-6

Vuong's non-nested test: Negative binomial vs zero-inflated negative binomial

Chasmogamous Bud Data			
	z-statistic	*H_A*	*p-value*
Raw	-4.70	model 2 > model 1	1.31e-6
AIC-corrected	-4.01	model 2 > model 1	3.09e-5
BIC-corrected	-2.94	model 2 > model 1	1.6e-3

Cleistogamous Bud Data			
	z-statistic	*H_A*	*p-value*
Raw	-7.50	model 2 > model 1	3.26e-14
AIC-corrected	-6.68	model 2 > model 1	1.22e-11
BIC-corrected	-5.42	model 2 > model 1	3.05e-8

Likelihood ratio test: Zero-inflated Poisson vs zero-inflated negative binomial

Chasmogamous Bud Data			
	Log-likelihood	*Chi-squared*	*p-value*
Zero-inflated Poisson	-316.78	-	-
Zero-inflated negative binomial	-230.30	172.96	<2.2e-16

Cleistogamous Bud Data			
	Log-likelihood	*Chi-squared*	*p- value*
Zero-inflated Poisson	-451.76	-	-
Zero-inflated negative binomial	-287.47	328.57	<2.2e-16

Akaike information criterion: All vs all

	Chasmogamous Data *AIC*	Cleistogamous Data *AIC*
Poisson	913.46	1768.74
Negative binomial	555.96	698.63
Zero-inflated Poisson	657.56	927.52
Zero-inflated negative binomial	486.60	600.94
